# The use of lateral flow lipoarabinomannan for diagnosis of TB in advanced HIV disease in Abia State, Nigeria

**DOI:** 10.4102/jphia.v16i1.1265

**Published:** 2025-08-06

**Authors:** George Ikpe, Chukwuebuka Ugwu, Chukwuemeka Amuta, Chibueze Oparaocha, Chika J. Anyigor, Peter I. Omoniyi, Okwudili Chukwudinma

**Affiliations:** 1 Department of Public Health, Federal Ministry of Health, Abuja, Nigeria; 2 Department of Public Health, Faculty of Community Medicine, Abia State University, Aba, Nigeria; 3 The National Tuberculosis, Buruli Ulcer and Leprosy Control Program, Department of Public Health, Federal Ministry of Health, Abuja, Nigeria; 4 Light Consortium, ECR, Liverpool School of Tropical Medicine, Liverpool, United Kingdom; 5 Zankli Research Center, Bingham University, Abuja, Nigeria; 6 Catholic Caritas Foundation of Nigeria, Umuahia, Abia State, Nigeria; 7 Department of Medical Records, Federal Medical Center, Umuahia, Nigeria; 8 School of Society, Community and Health, Bedfordshire University, Luton, United Kingdom; 9 Department of Planning, Reporting and Accountability, African Centers for Disease Control and Prevention, Abuja, Nigeria; 10 Department of Public Health, Nigerian Sustainability and HIV Impact Project, Abuja, Nigeria

**Keywords:** LF-LAM, active tuberculosis, advanced HIV disease, health workers, Abia State

## Abstract

**Background:**

Lateral flow lipoarabinomannan (LF-LAM) test used in the diagnosis of active tuberculosis (TB) among patients with advanced human immunodeficiency virus (HIV) disease remains a relatively new approach in the diagnosis of TB in Nigeria. This study focused on the use of LF-LAM assay Alere Determine™ in the diagnosis of active tuberculosis among patients with advanced HIV disease in Abia State.

**Aim:**

This study was carried out to identify potential gaps that could be missed along the LF-LAM implementation cascade, which can be strengthened to improve quality of patients’ care, while gaining insight into health workers’ understanding of the test.

**Setting:**

This study was carried out in Abia State, Nigeria.

**Methods:**

Electronic data were extracted through a query run on health facility electronic databases, while manual chart abstraction was performed in facilities without and incomplete electronic medical records. In addition, qualitative interviews were conducted among health workers to gain insight.

**Results:**

Out of 1249 newly enrolled patients who were eligible for the test, only 605 (48.4%) were tested, and 644 (51.6%) were missed within the study period (October 2022 – September 2023). Out of this number, 159 (26.3%) were positive for the test, and only 68 (42%) were sent for further testing with GeneXpert, while 30 (18.9%) had no testing with GeneXpert and 61 (38.9%) had no documentation.

**Conclusion:**

Low awareness and capacity among health workers including poor documentation practices contributed to missed opportunities for the patients who could have benefitted from this test.

**Contribution:**

The study recommends comprehensive training of healthcare workers on the utilisation of LF-LAM test and improvement of documentation practices in Abia State and Nigeria.

## Introduction

Despite being preventable and curable, tuberculosis (TB) remains a global public health burden and an infectious killer disease, with up to 10 million infections and 1.5 million deaths annually.^1^ It is the commonest cause of death among people living with human immunodeficiency virus (HIV) with an estimated 214 000 deaths in 2020, representing one-third of HIV-related deaths.^2^ Close to 22% of the total number of TB patients tested for HIV were co-infected.^2,3^ People living with HIV are more likely to develop TB than HIV-negative patients.^4^ Over two-thirds of HIV and TB related deaths are not reported because of underdiagnosis and regarded as one of the main challenges facing case detection in many TB programmes.^5^

The World Health Organization (WHO) End-TB strategy (2015–2030) lays emphasis on prompt diagnosis and treatment of TB among people living with HIV^6^ and improving access to prompt diagnosis of TB can make the difference in the fight against TB particularly among people living with HIV, which is a key component of the ‘End-TB strategy’. Diagnosis of TB among HIV-positive patients can be very challenging particularly among those with advanced disease, as studies have reported significant mortality rates of over 35% as a result of TB among people living with HIV.^5,7^ This is because they are very ill, and most times may not be able to produce sputum and chest X-rays appear atypical.^8^ It is also known that over 50% of hospitalised patients living with HIV and/or AIDS do not produce good sputum samples for analysis.^9^ Extra-pulmonary TB is also very common among this group of patients and use of sputum for diagnosis and chest X-rays may not be suitable. It is estimated that up to 47% of the cases of TB and HIV co-infection were missed mainly because of poor access to diagnostic tools.^6,10^

World Health Organization in 2015 released the first guidelines for the use of urine lateral flow lipoarabinomanan (LF-LAM) assays as point-of-care tests (POCs) for the diagnosis of active TB in patients with advanced HIV disease (AHD) and was later updated in 2019.^2^ Lipoarabinomannan (LAM) is a glycolipid present in the mycobacterial cell walls and released from degenerating or metabolically active mycobacterium tuberculosis (MTB) cells in patients with the active disease.^11^ It is an immune modulator and virulence factor used by MTB to inactivate macrophages and oxidative radicals for killing the organism.^12^ Lipoarabinomannan is also a prognostic maker and a predictor of risk of mortality, as studies have reported that mortality is over two times more in patients with a positive LAM assay than patients with negative LAM.^13^ Studies have demonstrated that routine LAM testing has led to a 4% absolute risk reduction in mortality.^14^

Following the conclusion of phase 1 implementation of LAM testing in 28 facilities across four states in Nigeria, Anambra, Lagos, Akwa Ibom, and Rivers in February 2021, using the lessons learned, The Federal Ministry of Health (FMOH) adopted the use of LF-LAM as a point-of-care tests for the diagnosis of TB inpatients with AHD (cluster of differentiation 4 [CD4] count of 200 cells/mm^3^ and below) or the in the ‘seriously ill’ – unable to walk unaided, fever of 39 degrees, respiratory rate of 30 cycles/min and heart rate of 120 beats (i)/min^5^.

This directive from the FMOH recommended that patients with positive LF-LAM results should be commenced on treatment for drug susceptible tuberculosis while sputum samples are collected for Xpert/Truenat examination.^15^

Following the FMOH adoption of LF-LAM test, which is relatively new in the HIV treatment programme, this study was carried out to identify potential gaps that could be missed along the LF-LAM implementation cascade, which can be strengthened to improve quality of patients’ care, while gaining insight into healthcare workers understanding, perception, impact, ease of use and confidence in the test results. The data and results from this study will be used to strengthen the LF-LAM implementation in Nigeria after it is shared with major stakeholders and policymakers in the FMOH. This we believe will lead to an improved AHD programme and consequently good patient outcomes.

## Research methods and design

### Design

This study utilised a mixed methods research approach of the concurrent nested variety comprising a cross-sectional programmatic assessment and a qualitative enquiry.^16^ The cross-sectional component represents a twelve-month snapshot of LF-LAM use in screening and diagnosing active TB among people living with HIV on antiretroviral therapy (ART) in Nigeria. The qualitative component comprised key informant interviews framed from a phenomenological standpoint conducted among healthcare professionals using LF-LAM.

To deepen our understanding of the application of urine LF-LAM assays as point-of-care tests for the detection of active tuberculosis in individuals with AHD, we carried out key informant interviews (KII) with the aim of gaining valuable insights and perspectives pertaining to this particular subject matter, which may not be captured in the quantitative component. The data collected were analysed by simple content analysis after transcribing the voice recording.

### Setting

This research was carried out in Abia State Southeastern Nigeria. With an estimated population of over 4.2 million by projection using the 2006 National Population Census. It has 17 LGAs and 192 political wards. According to the Nigerian acquired immunodeficiency virus (AIDS) Indicator and Impact Survey of 2018, the state has an HIV prevalence of 2.1 that is higher than the national prevalence rate of 1.4.^17^ The state has 38 HIV comprehensive ART health facilities across public and private providers rendering primary, secondary and tertiary care services, and 8 facilities were chosen for this study. The comprehensive ART facilities render the full complement of HIV-related services such as laboratory diagnostic and monitoring testing services, PMTCT services, paediatric ART services, adult ART services and other integrated services including TB and HIV collaborative care. This study involved comprehensive ART sites as it concerns the care of people with AHD in their care.

**FIGURE 1:**
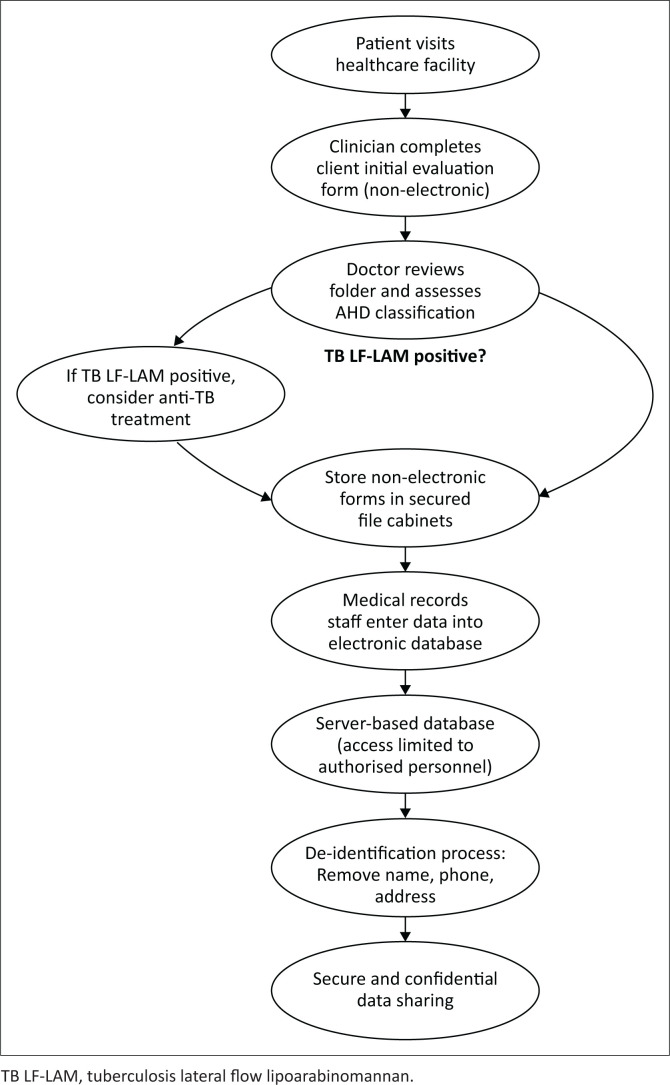
Data collection processes.

At the comprehensive ART sites, patient details are captured on both non-electronic and electronic record forms in the facilities. The electronic database is derived from the non-electronic records of patients. Entries regarding patient details – such as name, sex, age, family and personal medical history, and other biomedical parameters – are first captured in a non-electronic format. For new patients, this is referred to as the ‘client initial evaluation form’. It is completed by the clinician and indicates the key parameters for classifying a client as presenting with AHD or being severely ill. The CD4 test result, the WHO clinical stage, the TB LF-LAM result, and the physical signs observed and elicited during the examination are entered into this form. The forms are embedded in patients’ folders, which a doctor reviews to make decisions about commencing the patient on anti-tuberculosis treatment when the TB LF-LAM results are positive. Patients’ files are securely stored in file cabinet to limit access to unauthorised personnel within the record rooms of the healthcare facilities, see Figure 1.

### Sampling

A simple random sampling was performed for this study to select facilities. Eight comprehensive health facilities trained and supported to use LF-LAM for the diagnosis of TB were randomly sampled for this study. At each facility, clinical records of all people living with HIV diagnosed with AHD per national guidance were assessed for the use of LF-LAM for the screening of TB.^15^ The AHD refers to (CD4 cell count ≤ 200 cells/mm^3^) and WHO staging 3 or 4,^15,18^ or those who are diagnosed as being ‘severely ill’ – hyperpyrexia (temperature ≥ 39 °C), tachypnoea (respiratory rate ≥ 30 cycles per minute), tachycardia (heart rate ≥ 120 beats per minute), and inability to walk unaided formed the inclusion criteria for the people living with HIV. Any HIV-positive patient presenting with any of this symptom is classified as being severely ill and therefore eligible for the test.

To ensure objectivity by the attending clinician, the patient ‘client initial evaluation form’ contains the WHO different staging depending on the patient initial presenting symptoms or complains, the attending clinician can then stage the patient either as stage 1, 2, 3 or 4. For this study, any patient who has been staged 3 or 4 by the attending clinician is regarded as being eligible for the LF-LAM test. Dedicated hospital staff in the medical records departments upload the data from the forms of new clients to the electronic medical record database. These are server-based databases, with access granted only to these personnel and their supervisors. The data are coded so that individual patients cannot be identified by name, phone number or address if the database is shared – a process known as de-identification is carried out.

For the qualitative component, ten healthcare professionals trained for the use of LF-LAM across the selected facilities were purposively selected and interviewed as key informants. Participants comprised ART physicians, nurses, laboratory scientists, viral load champions which are lay workers some of whom are people living with HIV.

### Data collection and analysis

Lateral flow lipoarabinomannan and Xpert/MTB rifampicin (RIF) or Xpert Ultra test results of the included patients was abstracted using queries to ensure uniformity across the sites from the electronic records and client evaluation forms, while manual extraction was performed for facilities with incomplete documentation using standardised abstraction forms. Clinical symptoms data were obtained from the charts of all clients who met the stated inclusion criteria. In addition, data of laboratory investigations conducted for each patient were obtained from the laboratory register and electronic records of the selected health facilities in case of incomplete documentation. This became necessary because some of the patients had incomplete documentation and entry of their results and other clinical data including CD4 count results in their registers and electronic records.

**FIGURE 2:**
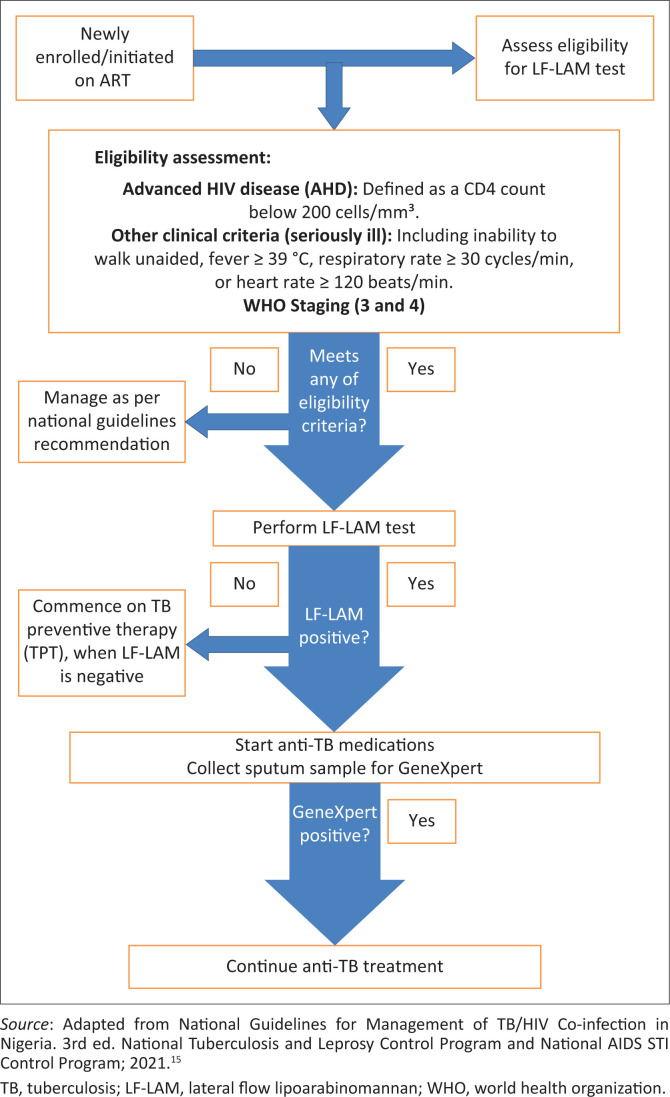
Lateral flow lipoarabinomannan cascade.

It should be noticed here that the cascade mentioned in this study refers to the algorithm developed by the FMOH in the guidelines for management of TB and HIV co-infection.^15^ The cascade begins with eligibility for the LF-LAM test either by CD4 count or ‘seriously ill’ among people living with HIV, followed by collection and testing of urine using the LF-LAM strip. For positive tests, they are commenced on anti-TB medications for drug sensitive TB, while sputum samples are collected and sent to Xpert MTB RIF, and based on the results, they can be started on medications for drug-resistant TB if the Xpert MTB RIF results are positive for rifampicin resistance, but if no resistance is detected or the result is negative, the patient is continued on the medications already started (see Figure 2).

The variables of interest in this study include the number of people living with HIV meeting the inclusion criteria for AHD, the number of patients evaluated for TB using LF-LAM, the number of patients positive for the LF-LAM test, the number of patients tested with Xpert MTB/RIF (or ultra), the number of patients diagnosed with TB, and the number commenced on TB treatment and the number of patients placed on TB preventive therapy (TPT). Simple proportions were reported for the percentage of clients completing each step of the cascade.

Ten healthcare professionals were purposively selected from the eight randomly selected hospital facilities included in this study in Abia State to participate in the KIIs.

The qualitative interactions were guided by an iteratively developed interview guide using a semi-structured style, which gave opportunity for probing in the direction of the participants’ narrative in line with the interpretative approach to the research. All interviews were audio recorded and transcribed verbatim. Transcripts were analysed using the framework thematic analysis approach conducted first by hand and then aided by Microsoft Excel^®^ software version 2016. Labelling, indexing, and identification of themes and representative quotes were carried out in group by the multidisciplinary research team of C.U., G.I., A.I., C.O. and A.O.C. in reflexive over a period of four weeks. Theme quotes are presented as they relate to each other and to the objective of the study.

### Ethical considerations

Ethics approval for this mixed methods research was obtained from the Research and Ethics Unit from the Directorate of Public Health and Disease Control of the Abia State Ministry of Health with approval number: ASMH/EC/22/010.

## Results

The age and sex distribution of the patients enrolled in ART is presented in Table 1. The findings indicate that a total number of 4068 individuals were registered in the ART programme during the study period, with males accounting for 55.0% of the patients, 45.0% of the total population being females. Similarly, the 30–45 year age group with 33% constituted the majority of people living with HIV on ART.

**TABLE 1:** Background information on enrolled patients (*N* = 4068).

Participants information	Number enrolled on ART	%
Number eligible for LF-LAM	1249	31.0
**Eligibility criteria**		
WHO staging (3 and 4)	250	20.0
CD4 Count < 200 cells/mm^3^	32	3.0
Seriously ill	967	77.0

ART, antiretroviral therapy; LF-LAM, lateral flow lipoarabinomannan; WHO, World Health Organization; CD4, cluster of differentiation 4.

A total number of 1249 patients were identified as being eligible for LF-LAM test using CD4 count test, ‘other clinical criteria’ and WHO clinical staging as per our national guidelines for LF-LAM test eligibility.^15^ Among these, 449 patients, accounting for 36% of the overall population, were males, while 64% were females, see Table 2. It was observed that some of the patients who are termed as ‘seriously ill’ had CD4 count more than 200 cells/mm^3^ and were in either WHO stage 1 or 2, whereas one would have expected that patients with lower CD4 count would have been in the seriously ill category and WHO stage 3 or 4 disease classification. So there was overlap for the criteria for AHD identification and eligibility for the LF-LAM test using the different criteria, with some of the patients also being seriously ill, and having CD4 count of less than 200 cells/mm^3^, and WHO stage 3 and 4.

**TABLE 2:** Information on patients with referred for lateral flow lipoarabinomannan.

Participant information	Number of patients eligible for LF-LAM (*n* = 1249)	%
**Patient LF-LAM testing status (*n* = 1249)**		
Patient with LF-LAM result	605	48
Patient without LF-LAM result	644	52
**LF-LAM positivity status (*n* = 605)**		
Positive	159	26
Negative	446	74
Total number of patient commenced on anti-TB medication (*n* = 159)	88	55
Total number of patients commenced of TB preventive therapy (TPT)	0	-

LF-LAM, lateral flow lipoarabinomannan; TB, tuberculosis.

Using the eligibility criteria, only 605 (48%) eventually had the test with results showing 26% (*n* = 159) positivity rate among patients. Out of this number, only 88 (55%) were commenced on first line anti-TB medications as per the national guidelines, revealing many missed opportunities to improve diagnosis and outcome of the patients.

### Qualitative findings

We carried out key informant interviews on 10 healthcare professionals who were purposively selected from the 8 randomly selected hospital facilities, see Table 4. The participants had varied perceptions of the LF-LAM test in terms of understanding, ease of use, confidence, impact and feasibility of the results. The findings from the interview are presented below.

### Perception outcomes for key informant interview on the use of urine lateral flow lipoarabinomannan assays as point-of-care tests for the diagnosis of active tuberculosis in patients with advanced HIV disease

#### Understanding of the lateral flow lipoarabinomannan test and its use

The results from the interviews indicate that all of the respondents (100%) were already aware of LF-LAM. Among the respondents, while some learned about LF-LAM by reading about it, hearing about it from colleagues, others heard about it from their institution. Some healthcare workers (HCWs) also attended a training about LF-LAM conducted by an implementing partner supporting HIV care and treatment services.

In addition, two-fifths of the respondents have used or requested LF-LAM, while 20% have not used it but have observed their colleagues using it. This indicates an increased utilisation of LF-LAM among the respondents. In terms of ease of use, 90% of the respondents stated that it is very easy to obtain a valid result using LF-LAM, while 10% found it challenging because basic laboratory skills are required.

Regarding the diagnostic guidelines by the National Tuberculosis, Buruli Ulcer and Leprosy Control Programme (NTBLCP) for LF-LAM application, three-fifths of the individuals surveyed believe that the algorithm is easily accessible, user-friendly, and adheres to appropriate standards. Nevertheless, 20% of the respondents are unaware of the algorithm’s existence because of its limited accessibility in the health facilities, excluding laboratory professionals, which makes it challenging to understand. One interviewee shared the following insight:

‘I learned about LF-LAM from my colleagues at work when I saw them using it. It’s a great innovation and very user-friendly. It has made detecting TB patients much easier. It is easy to understand and to use. The algorithm is easily accessible, user-friendly, and follows the right standards.’ (Female, ART nurse, 8 years of providing care)

#### Ease of use of lateral flow lipoarabinomannan test

The results of the survey show that most participants find interpreting the LF-LAM examination results easy. On average, more than half of the respondents think the LF-LAM guideline is easily accessible and can effectively detect TB in HIV-positive individuals with low CD4 cell counts. The majority of respondents reported that following the LF-LAM guideline is very easy, but some find it challenging. About half of the respondents are aware of the follow-up test for people living with HIV who test positive with LF-LAM using Xpert MTB/RIF, which helps to identify or rule out rifampicin-resistant Tuberculosis. The participants generally find the eligibility criteria for LF-LAM usage satisfactory and easy to understand. However, there were concerns about limiting LF-LAM usage to AHD patients, although some believe that it may be challenging to detect tuberculosis among those without symptoms. One interviewee shared the following insight:

‘The LAM test result is easy to interpret, although some HCWs lack the capacity to follow the LF-LAM guideline the way it ought to be followed.’ (Male, laboratory scientist, 5 years of providing care)

#### Confidence in lateral flow lipoarabinomannan test results

The level of confidence of healthcare workers on LF-LAM results was mixed. While some trusted the test as a guide for clinical management others were not willing to base the clinical treatment of a patient solely based on the test citing the risk of false positives. On the other hand, participants reported that patients generally had confidence in the test result and were willing to be treated for TB based on it.

#### Impact and feasibility of lateral flow lipoarabinomannan test results

The impact and feasibility of LF-LAM test results were examined. All the respondents believed that the test improved patient management by enabling prompt diagnosis and treatment. Using LF-LAM has a major advantage: it is faster than Xpert MTB/RIF, meaning more patients can be tested. One interviewee shared the following insight:

‘The LF-LAM test is reliable, with easy sample collection and consistent results when compared to Xpert MTB RIF. The test can be readily performed in settings lacking a GeneXpert machine, thereby enabling the prompt initiation of patients’ treatment.’ (Male, Viral load champion, 2 years of providing care)

The LF-LAM test has some drawbacks, like less depth and sensitivity compared to Xpert MTB RIF, as the sensitivity increases with decreasing CD4 cell count clients, inability to detect drug-resistant TB.^2^ Many health workers lack knowledge on using LF-LAM for TB diagnosis. Some respondents faced challenges like lack of awareness, inconsistencies compared with Xpert MTB RIF results, and reluctance to rely solely on LF-LAM for diagnosis. Suggestions for expanding LF-LAM use included using it for all patients with symptoms of tuberculosis regardless of HIV or AHD status, ensuring personnel are trained, and availability of LF-LAM test kits. One interviewee shared the following insight:

‘Most health workers in Abia State lack extensive knowledge on using LF-LAM for TB diagnosis. Some are unaware that LF-LAM testing is done at service delivery points and some HCWs don’t trust LF-LAM test results without seeking confirmatory GeneXpert testing. There is need to train HCW’s in Abia State very well on the use of LF-LAM if the battle for curbing the spread of TB is to be achieved in the state.’ (Male, laboratory scientist, 7 years of providing care)

The respondents shared their experiences about the ease of access and availability of LF-LAM test commodities. Some respondents found it easy to access, while others believed it’s not easy to access and most times unavailable. Some respondents reported no stock-outs of LF-LAM test kits, while others experienced stock-outs once or for some time. The use of LF-LAM has positively influenced the management of people living with HIV and AHD.

However, there are concerns about its limited use and poor knowledge among healthcare workers. The LF-LAM testing implementation has been relatively easy, with some concerns about awareness among clinicians. Some healthcare workers lack trust in relying solely on LF-LAM results without further testing with Xpert MTB RIF and commencement of patients on anti-TB medications. Some hospital facilities in Abia State have experienced stockouts and poor access to LF-LAM testing kits. Addressing these areas of need will enhance preference for and reliance on LF-LAM test results by healthcare workers in Abia State.

## Discussion

As shown in Table 2, 26% positivity rate was observed among patients who were tested using the LF-LAM test at the ART clinic. Similar findings were observed by Bjerrum and her colleagues in 2019.^19^ From Table 3, a greater proportion (77.4%) of those who were eligible for LF-LAM test had CD4 count less than 200 cells/mm^3^, according to WHO definition for AHD and therefore recommended a package of care for such patients, which included screening, management and prophylactic treatment of opportunistic infections such as TB, cryptococcal meningitis, toxoplasmosis, et cetera.^20^

**TABLE 3:** Information of patients with lateral flow lipoarabinomannan positive results.

Participant information	LF-LAM positive result (*n* = 159)	%
**LF-LAM results based on eligibility criteria**		
WHO staging (stage 3 and 4)	32	20.1
CD4 count < 200 cells/mm^3^	123	77.4
CD4 count > 200 cells/mm^3^ (seriously ill)	4	2.5
**GeneXpert test status**		
Sample sent for GeneXpert	68	43.0
Sample not sent for GeneXpert	30	19.0
Unknown/no documentation	61	38.0
**Patients MTB status (*n* = 68)**		
Positive	40	58.8
Negative	28	41.2

LF-LAM, lateral flow lipoarabinomannan; MTB, mycobacterium tuberculosis; WHO, World Health Organization.

Over 30% of the patients who were eligible for the test were based on the WHO clinical stage 3 and 4, which is used for surveillance purposes. Nevertheless, use of this method for AHD diagnosis has been found to have reduced specificity for prediction of patients with CD4 count of less than 200 cells/mm^3^ and therefore many patients who could have benefitted from the test and other package of care were missed.^21,22^ Less than half of the patients (48%) who were eligible for the test were tested for LF-LAM, there could be a possible selection bias that could have led to this results, but we also think that that this may have been because of a lack of capacity on the part of the clinicians or other healthcare workers to order for the tests for those who are eligible.

Only 2.5% of the patients with CD4 greater than 200 cells/mm^3^ had a positive LF-LAM test. These results are not surprising because it is known that the sensitivity of LF-LAM results increases with decreasing CD4 counts and vice versa.^9^ There is a possibility that most health workers, especially clinicians, do not know about the ‘other clinical criteria’ for patients whose CD4 count may be above 200 cells/mm^3^ of blood. These include being unable to walk unaided, fever greater than 39 degrees and above, heart rate of 120i/minute and respiratory rate of 30 cycles per minute and above and hence were not sent for the LF-LAM test.

Furthermore, only 43% of the positive samples were sent for Xpert MTB/ RIF assay following our national guidelines for LF-LAM testing. The remaining 19% (*n* = 30) and 38% (*n* = 61) of samples were not sent for further evaluation and had no documentation, respectively, highlighting not only missed opportunities to improve quality in patient management and outcomes but also documentation gaps in the health facilities. The huge documentation gaps were noticed mostly in the laboratory registers, where either the viral load champion or the record officer forgot to enter the patients results into the registers and then into the electronic storage. Other times, there will not be any documentation as to whether an eligible patient for LF-LAM has been sent to the GeneXpert laboratory by the attending clinician.

**TABLE 4:** Qualitative key informant interview participants.

Category	Sex disaggregation (*n*)	Years providing HIV care (years)
**ART physicians**	-	3–15
Males	2	-
Female	1	-
**ART nurses**	-	4–11
Males	0	-
Females	2	-
**Laboratory scientist**	-	2–9
Males	2	-
Females	1	-
**Viral load champions**	-	1–2
Males	1	-
Females	1	-

HIV, human immunodeficiency virus; ART, antiretroviral therapy.

It was noticed by one of the respondents that some of the healthcare workers lack the capacity to follow the algorithm rightly. It was inferred from the interview that about half of the respondents were either not aware of further testing with Xpert/MTB/RIF, or lack the capacity, which could have affected their not requesting for further evaluation of sputum samples from patients, representing a major opportunity missed for patient management. Almost 60% of the positive LF-LAM samples sent for Xpert MTB/RIF came out positive as mycobacterium tuberculosis using sputum. Similar results were also found by Shah et al. in 2014.^23^ This is usually performed to rule out rifampicin-resistant tuberculosis as LF-LAM testing does not have the capacity to identify drug-resistant TB. We also observed that 74% (*n* = 446) of the patients who had negative LF-LAM results and were eligible for TB preventive therapy (TPT) were not commenced on it, and it was a missed opportunity to improve the outcome of patients with AHD. The TB preventive therapy has been shown to significantly improve the health and survival of people living with HIV by reducing the risk of tuberculosis.^24^

From the KII carried out, it was observed that the workplace was the primary source of knowledge about the use of LF-LAM among healthcare workers as majority of healthcare workers have not been formally trained on the LF-LAM assay as of the time of this study. From the key informant interviews, we observed that healthcare workers in Abia State found LF-LAM easy to understand and used it for obtaining valid results. This has the potential to significantly influence TB control efforts, particularly in high-HIV-burden settings.

There was mixed response with regard to confidence in the test results, while some had some level of confidence on the test results, others expressed concerns about using only the results for commencement of anti-TB medications. This further highlights sub-optimal knowledge of healthcare workers about the LF-LAM test. Regarding the impact and feasibility of test results, all respondents believed that LF-LAM results improved patient management and patient outcomes because of quick application and produced results in less than 30 min.^9^

## Conclusion and recommendations

This study focused on the implementation of LF-LAM as a point-of-care test in the diagnosis of active tuberculosis among patients with AHD in Abia State, looking at opportunities that were missed along the implementation cascade. There were many missed opportunities to improve the quality of patient care and outcomes along the cascade of care for people living with HIV eligible for the LF-LAM test, TB preventive therapy and poor documentation. While healthcare workers in Abia State find it easy to use LF-LAM for TB diagnosis, there is a lack of awareness and understanding of LF-LAM test among them. There were also challenges regarding confidence in LF-LAM test results among healthcare providers in Abia State including inconsistencies with Xpert MTB RIF results, reluctance to rely solely on LF-LAM, limited sensitivity of LF-LAM compared to Xpert MTB RIF test.

This study advocates for comprehensive training of healthcare professionals such as doctors, nurses, laboratory scientists and records officers in Abia State and the country on the utilisation of LF-LAM test in the identification of active tuberculosis among patients with AHD in Abia State. In addition, there is a need for improvement in documentation of patients’ records both in the patient registers and in the electronic records by healthcare workers in the facilities.
